# Label-Free Long-Term Methods for Live Cell Imaging of Neurons: New Opportunities

**DOI:** 10.3390/bios13030404

**Published:** 2023-03-20

**Authors:** Zrinko Baričević, Zahra Ayar, Samuel M. Leitao, Miranda Mladinic, Georg E. Fantner, Jelena Ban

**Affiliations:** 1 Department of Biotechnology, University of Rijeka, Radmile Matejčić 2, 51000 Rijeka, Croatia; zrinko.baricevic@uniri.hr (Z.B.); mirandamp@uniri.hr (M.M.); 2 Laboratory for Bio- and Nano-Instrumentation, Institute of Bioengineering, School of Engineering, Swiss Federal Institute of Technology Lausanne (EPFL), 1015 Lausanne, Switzerland; zahra.ayar@epfl.ch (Z.A.); samuel.mendesleitao@epfl.ch (S.M.L.)

**Keywords:** neuronal cultures, live cell imaging, label-free imaging, phototoxicity, scanning ion conductance microscopy, atomic force microscopy, digital holography microscopy

## Abstract

Time-lapse light microscopy combined with in vitro neuronal cultures has provided a significant contribution to the field of Developmental Neuroscience. The establishment of the neuronal polarity, i.e., formation of axons and dendrites, key structures responsible for inter-neuronal signaling, was described in 1988 by Dotti, Sullivan and Banker in a milestone paper that continues to be cited 30 years later. In the following decades, numerous fluorescently labeled tags and dyes were developed for live cell imaging, providing tremendous advancements in terms of resolution, acquisition speed and the ability to track specific cell structures. However, long-term recordings with fluorescence-based approaches remain challenging because of light-induced phototoxicity and/or interference of tags with cell physiology (e.g., perturbed cytoskeletal dynamics) resulting in compromised cell viability leading to cell death. Therefore, a label-free approach remains the most desirable method in long-term imaging of living neurons. In this paper we will focus on label-free high-resolution methods that can be successfully used over a prolonged period. We propose novel tools such as scanning ion conductance microscopy (SICM) or digital holography microscopy (DHM) that could provide new insights into live cell dynamics during neuronal development and regeneration after injury.

## 1. Introduction

The nervous system is one the most complex and sophisticated biological structures. Neurons, in tight coordination with glial cells, represent the functional units of the central nervous system (CNS) and are responsible for information transmission, processing and coding, allowing for the exceptional cognitive abilities such as language, memory, abstract thinking and reasoning [1,2].

Neurons are extremely polarized cells, meaning that their shape is highly asymmetric and organized into structurally and functionally specialized domains, more commonly consisting of one thin and long axon and many thicker and shorter dendrites extending from the neuronal cell body [3,4,5,6]. The length of an axon can exceed the cell body diameter by several orders of magnitude, and this requires an efficient transport machinery to allow long distance signaling [7,8].

Another fundamental characteristic that makes neurons a not-expandable cell source is that once they complete neurogenesis, they become postmitotic cells and continue to suppress the cell cycle throughout their lifetime [9,10]. Moreover, the mature mammalian CNS neurons (unlike the peripheral neurons and the CNS of lower vertebrates) fail to (fully) regenerate following injury, and the exact reasons and advantages for this evolutionary choice in turning off regeneration and plasticity in the adult mammals are still poorly understood [11,12,13,14]. A full description of the molecular mechanisms that occur during development and maturation of such a complex system is fundamental for designing effective therapies for neurodegenerative diseases, since many common developmental pathways, including cell cycle machinery, are found to be misexpressed in Alzheimer’s disease (AD) and amyotrophic lateral sclerosis (ALS) patients [2,10,15,16].

The establishment of the neuronal polarity, i.e., axon-dendrite specification, was described in 1988 by Dotti, Sullivan and Banker [17]. The findings were so clear and consistent that this paper continues to be cited 30 years later [4]. Even though they did not have automated and sophisticated imaging devices that are available to researchers today, Dotti et al. described five morphological stages that occur during axon and dendrite specification of hippocampal neurons, using a 35 mm camera and videotape recorder as imaging devices and a hair dryer as a temperature controller [4]. Importantly, neuronal polarization and many other phenomena including axon guidance, synaptogenesis, neuronal network formation, maturation and activity were successfully recapitulated in vitro and also confirmed in vivo [18,19]. This and numerous works in the following years showed that even in a simplified format, we can (to some extent) model the CNS in vitro, significantly reducing or avoiding in vivo animal testing [20].

In vitro cell models, together with the huge technological advancements in recent years, offer numerous tools to obtain sophisticated imaging, measurements and analyses aiming to elucidate molecular mechanisms of neuron physiology including axon growth, cytoskeletal organization, synaptic activity, and many others. For instance, the periodic (~180–190 nm) ultrastructure of axons made by actin rings in association with spectrin, adducin and other proteins was discovered using super-resolution fluorescence microscopy methods [21,22]. In particular, live cell imaging provides insight into the dynamics of cellular events and overcomes the fixation-induced artifacts [23,24]. However, most live cell imaging methods rely on labelling of the specific cell biomolecules, which often interferes with cell physiology and experimental setting by causing damage and premature cell death.

In this paper we will review the fundamental requirements for optimal preparation, maintenance and imaging of (2D) neuronal cultures in vitro. Advantages and drawbacks of the labelling-based approaches will be discussed. The appropriate choice of cell source, media, culture conditions and imaging techniques will be described and discussed, with particular emphasis on label-free methods for observing live, unfixed cells with improved resolution using emerging techniques such as scanning ion conductance microscopy (SICM), atomic force microscopy (AFM) and digital holography microscopy (DHM).

## 2. Cell Source

### 2.1. Primary Neuronal Cultures

Neuronal cultures are among the most challenging biological samples to prepare and maintain in vitro. The main obstacle is the postmitotic state of neurons that does not allow their expansion. In addition, preparing primary cultures directly from the living nerve tissue implies their limited lifespan and their decreased survival with increasing age. For this reason, primary neuronal cultures are mostly prepared from late embryonic or early postnatal brain regions such as the cortex, hippocampus or cerebellum [25,26,27]. Spinal cord primary cultures [28,29,30,31] are less frequently used, but recently, more effective protocols with extensive cell characterization have become available [32,33,34,35]. Among mammalian species, rodents (mice and rats) are used predominantly, but primary cultures from other mammals such as opossums [36,37], pigs [38], sheep [39] and monkeys [40] have also been established. Inter-species variabilities can, for many aspects including adult neurogenesis, be a cause of misinterpretation in translation of the obtained knowledge to humans [2,12,41], thereby necessitating the development of a wider source of mammalian CNS cells.

The main steps of the primary neurons’ preparation protocol consist of tissue isolation (dissection) and enzymatic and mechanical tissue dissociation followed by plating on pre-coated surfaces. Detailed protocols with a few variations have been available for decades [25,26] and were further improved over time, leading to long-term cultures that can efficiently maintain neurons for several months in vitro [42,43,44,45]. This is of particular importance for modelling neurodegenerative diseases where aging is one of the most critical risk factors [46].

The main differences between available protocols include the use of different surface-coating molecules such as poly-L-lysine [25,26,47], poly-D-lysine [42,45,48] or poly-L-ornithine [36,43] for efficient cell adhesion. Extracellular matrix proteins such as laminin, collagen or fibronectin used both in isolated and purified [36,48] or a cell-secreted formulation such as Matrigel [43,49,50] can be subsequently used in combination with coating molecules, accelerating neurite outgrowth and improving neuronal survival [19,48,51]. Synthetic hydrogels such as PuraMatrix [44] are examples of the transition from the flat to the 3D substrates. While 3D substrates offer intriguing opportunities, we will focus on traditional 2D cultures in this review, since the flat substrates are ideally suited for high resolution, live cell imaging [18]. Nevertheless, it should be kept in mind that 3D neuronal in vitro models represent a rapidly growing in vitro platform (for a systematic review see [51]), even though they are much more challenging to prepare, manipulate and image. Another variation in experimental protocols during tissue dissociation steps includes both mechanical and enzymatic treatment with trypsin [25,26,36,45,47,48] or papain [42], in combination with DNase I [25,26,36,45]. Finally, the most frequently used maintenance medium is Neurobasal/B27 medium [25,26,36,43,47,48,50,52]. Alternatively, the enriched version, NbActiv4 (formulated by the addition of creatine, cholesterol and estrogen) [53], in combination with a commercially available astrocyte-conditioned medium (ACM) [42] can be used for long-term cultures.

Cell density is critical for neuronal survival. Neurons suffer at low density due to the significantly reduced cell–cell interactions. In vitro maturation, synaptogenesis and network activity are density-dependent [19,26,54,55]. When experimental conditions require isolated cells, paracrine trophic support that enhances neuronal survival can be provided by astrocytes, either in coculture with neurons plated directly on astrocyte monolayer [55] or suspended in a “sandwich” configuration, with neurons “hanging” above a monolayer of astrocytes [26]. Trophic support can be also provided using an astrocyte-conditioned medium (ACM) that contains several growth factors, signaling molecules, lipids, etc., secreted by astrocytes. Such a medium can extend the neuronal survival for weeks or even months [42,43,56]. Alternatively, serum-free low-density cultures in the absence of a glial cell feeder can be efficiently maintained for long durations, i.e., more than two [44] or three [45] months in vitro, allowing investigations of neuron-specific mechanisms.

A wide range of cell densities for primary neuronal cultures has been reported, from ultra-low (~2000 cells/cm^2^) [45], low (between 6500 and 8900 cells/cm^2^) [25,26,44], mid (between 26,000 and 80,000 cells/cm^2^) [36,50] to high density (up to 250,000 cells/cm^2^) [45,54]. However, the definition of low, medium and high density can be relative since in some studies three different plating densities correspond to different values. For example, low/medium/high densities correspond to 90,000, 180,000, and 360,000 cells/cm^2^, respectively, in one study [47] while in another study they correspond to 26,000, 52,000 and 263,000 cells/cm^2^ [55].

Unlike neurons, primary glial cells continue to divide in vitro, in the presence of growth factors. In particular, primary astrocyte cultures can be expanded (however, no more than two passaging are recommended) [57] and cryopreserved [26]. In addition to trophic support for low-density neuronal cultures, the primary cultures of astrocyte are increasingly used to investigate their role in many vital CNS functions. Excellent reviews of primary astrocytes are available [58], including an extensive list and comparison of the existing protocols taken from over 100 papers [59] and the more recent ones [60]. Primary microglia cultures are included as well in many of the forecited protocols [59,61].

Embryonic CNS tissues yield nearly pure neuronal cultures (~90% of neurons), while postnatal tissues result in mixed cultures with an increasing percentage of non-neuronal cells (astrocytes, microglia and oligodendrocytes) [25,26]. Purification methods such as immunopanning or shaking are used to separate different cell types [58]. Alternatively, to obtain microglia-free astrocytes, in vitro differentiation of neural stem cells (NSC) is used [62]. The coexistence of both neurons and glia does not necessarily represent a limitation; rather, they allow cell crosstalk (neuron–glia and neuron–neuron), more closely mimicking the in vivo context [47,54,55]. Indeed, specific protocols have been developed for co- and/or tri-cultures [63] and these can be used for neuroinflammation, and de- and regeneration studies.

### 2.2. Neuronal Cell Lines

To overcome the problem of limited cell numbers, immortalized (or secondary) cell cultures derived from neuronal and other tumors such as rat adrenal pheochromocytoma (PC12 cell line) [64] and malignant pluripotent embryonal carcinoma (NTERA-2 cell line) [65] were established. Neuroblastoma cell lines from human origin such as SH-SY5Y [66] are often used as in vitro neuronal models, offering an expandable, continuous and homogeneous source of cells, minimizing the variability between cultures and offering the possibility for a variety of experimental manipulations, including high-throughput screenings [19,67,68]. In addition, compared to the primary neurons, cell lines are easier to transfect [67]. However, important issues regarding their biological relevance must be considered. First of all, they carry genetic alterations [69] and during prolonged culture are subjected to passage-induced mutations [70]. Next, their phenotype remains relatively immature [67], although some recently improved differentiation protocols have been proposed [68]. Finally, functional issues must be considered, particularly regarding neurotoxicity studies, for which those cells were frequently used. For instance, four tested PC12 cell lines were found not to express functional NMDA receptors [71], while Neuro-2a cells showed much lower sensitivity to neurotoxins compared to primary neurons [72]. These and other discrepancies with the primary cell lines must be considered while designing experiments with immortalized cell cultures.

### 2.3. In Vitro Stem Cell-Derived Neurons

Another expandable, but more expensive and complex solution, is offered by stem-cell-based approaches. Pluripotent embryonic stem cells (ESCs) [73,74], induced pluripotent stem cells (iPSCs) derived from somatic cells such as fibroblasts [75,76], and multipotent neural stem cells (NSCs) that can be isolated from the adult brain [77,78,79] represent the expandable alternative since the induction of neural differentiation occurs entirely in vitro starting from isolated progenitor cells. Alternatively, the direct conversion of somatic cells into induced neurons (iNs) is also possible [80]. The main advantage of the cell reprogramming approach is the human cell source combined with the possibility to obtain patient-derived neurons that become a promising tool for disease modelling, bypassing ethical issues (no need for experimental animals and/or manipulations involving human embryos). However, time-consuming protocols, high costs and inefficient neuronal differentiation are still limiting their use [81].

It is important to keep in mind that when the stem-cell-based approach is used (and to some extent using neuroblastoma cell lines), the neuronal differentiation is induced in vitro (these neurons are “born” in vitro), while primary cultures derived from late embryonic or postnatal CNS tissue are mainly composed of already differentiated, post-mitotic neurons that upon dissociation regrow and “re-polarize” their processes [6].

**Figure 1:**
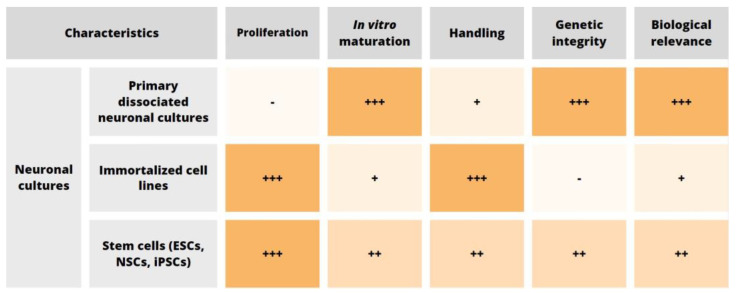
Neuronal in vitro models’ comparison. Neuronal cultures are classified according to their cell source (rows) and main characteristics (columns). The lack of proliferation in primary dissociated neuronal cultures refers to neurons and terminally differentiated glial cells (since glial cells also retain in vitro proliferation ability). Handling refers to ease of use in terms of culture preparation and maintenance procedures. - no; + low; ++ intermediate; +++ high level.

The advantages and disadvantages of the 2D neuronal in vitro models described before are summarized in Figure 1.

We can conclude that primary neurons remain the most biologically relevant cell choice for traditional 2D in vitro neuronal cultures, given their genetic integrity and the higher degree of morphological and physiological similarity with CNS tissue compared to the immortalized cell lines. Nevertheless, the progress made in enhancing the in vitro neuronal differentiation efficiency of human cells using reprogramming-based approaches could make them favorable candidate for biomedical studies. In addition, extensive comparative analyses are needed to fully characterize structural and functional properties of in vitro-generated neurons.

### 2.4. Neuronal Cell Death In Vitro

Neurons are extremely susceptible to any disturbance and show high phototoxicity [82,83], mechanosensitivity [84,85] and sensitivity to physical conditions (e.g., pH, temperature, etc.) [86]. To prevent their death, extreme care must be taken during in vitro procedures.

Among the many ways in which the cells may die, apoptosis and necrosis are considered predominant types of neuronal cell death [87,88]. However, recently it became evident that many more (“at least a dozen”) distinct mechanisms of cell death, particularly of neuronal death, exist, such as parthanatos, autophagic cell death, ferroptosis and many others, with distinct temporal, morphological, biochemical, and gene expression characteristics [87,89]. Often the different mechanisms of cell death present some overlapping characteristics, with similar or different molecular pathways involved. Moreover, the traditionally distinguished apoptosis and necrosis are now considered an apoptosis–necrosis cell death continuum in which neuronal death can result from varying contributions of co-occurring apoptotic and necrotic mechanisms [88]. It is particularly useful to use neuronal cell cultures to study molecular mechanisms of cell death induced by different insults such as acute oxidative stress, growth factor withdrawal, DNA damage, mechanical stress, etc. However, the potential source of damage and cell death can be due to the in vitro experimental conditions, in particular phototoxicity induced by imaging. Namely, fluorescence excitation causes phototoxicity to tissues and cells [90,91]. The main cause of phototoxicity in living cells is the oxygen-dependent reaction of free-radical species, which are generated during excitation of fluorescent proteins or dye molecules with surrounding cellular components. It is to be further investigated which molecular pathways may be involved in neuronal cell death in vitro, often involving irregular plasma membrane blebbing, visible large vacuoles or detachment from the tissue culture plate [92]. However, the protocols that avoid the detrimental effects of the light or other toxic in vitro parameters are preferable and provide the new possibility of analyzing fully functional neurons in their more physiological state.

We will not go through all the different cell death mechanisms that occur in vivo; rather, we will consider the potential source of damage and cell death due to the in vitro experimental conditions, in particular phototoxicity induced by imaging.

## 3. Label-Free Live Cell Imaging—Key Experimental Settings

Transmitted light (brightfield) microscopy suffers from low contrast since cells are transparent. Even with contrast-generating techniques such as phase contrast or differential interference contrast (DIC), the lateral and axial resolution in particular remain relatively low, limited to 200–500 nm [93,94].

The advantage offered by fluorescent labelling of live cells is unquestionable, given the targeted visualization of specific cellular compartments with a high signal-to-background ratio [91]. Membrane labelling with liphophilic tracers such as DiI [95] or lectin fluorescent dyes such as wheat germ agglutinin (WGA) have been used both in vitro and in vivo to trace the neurons [96]. Green fluorescent protein (GFP) and its numerous variants (with emission colors ranging from cyan to red) expressed intracellularly as fusion proteins with the protein of interest have been massively employed in cell biology [97,98,99,100]. However, this approach has several drawbacks. First, the expression of GFP-labelled proteins requires transfection, and primary neurons are notoriously difficult to transfect [101]. Second, transfection can lead to the overexpression of the protein of interest, in particular when strong promoters such as cytomegalovirus (CMV) are used [99]. Third, GFP and its derivatives are relatively big tags and for instance, when used to label actin, were shown to alter cell migration, response to mechanical stress, neuronal polarization and cell adhesion, significantly altering the cell physiology [102,103,104]. Finally, rapid photobleaching strongly reduces the available tracking/imaging time interval [92], although more photostable fluorescent protein variants are now available [98,99]. The need for transfection and GFP-based fusion proteins has been recently eliminated by the development of cell-permeable silicon-rhodamine (SiR) fluorescent probes for actin and tubulin [105].

Once suitable neuronal cultures are prepared, several experimental conditions should be controlled during imaging, to perform long-term experiments investigating cell growth, migration or regeneration. Temperature, humidity and CO_2_ must be precisely and continuously monitored, and this can be accomplished by using cage or top-stage incubators mounted on microscopes (both home-made and commercially available incubation systems can be used) [92]. Top- and glass-bottom dishes, multiwell slides and plates with compatible microscope stage inserts allow cell visualization and imaging while keeping the system “closed”, preventing contaminations. If label-free imaging is performed, the autofluorescence of growth media components such as riboflavin, folic acid [18] or phenol red used as a pH indicator is no longer an issue. pH is typically buffered by the addition of bicarbonate and HEPES to the medium [92]. In such a way, complete media formulations can be used without changing the conditions of the cell growth, which is essential for maintaining long-term cell viability. This is of particular importance since neurons are very susceptible to media exchange: frequent (i.e., daily) exchange of cell medium increases cell death [4]. Usually, neuronal cultures are maintained by changing only half of the volume twice per week [25,36,43] or even less frequently [26,45].

Biological relevance is one of the most important parameters to consider, and therefore primary dissociated neuronal cultures still represent the most efficient compromise between immortalized cell lines (that allow easy maintenance and expansion, high-throughput analysis, etc.) and in vivo experiments on animals.

## 4. Label-Free Methods for Long-Term and High-Resolution Imaging of Neurons

### 4.1. Digital Holography Microscopy

Digital holography microscopy (DHM) is a quantitative phase microscopy (QPM) technique enabling noninvasive, label-free optical imaging of transparent specimens such as living cells. DHM, compared to the fluorescence microscopy, exhibits lower phototoxicity and no photobleaching, and it is rapidly evolving with its applications in biomedicine [93,106]. DHM is based on the difference in the refractive index of specific cellular substructures. For instance, actin and tubulin fibers have higher density and therefore higher refractive indices compared to the cytoplasm. This induces a phase shift (delay) on the transmitted light wavefront, which is recorded as intensity variation on the hologram and can be reconstructed. Compared to conventional optical techniques such as phase contrast and DIC, DHM provides nanometric axial sensitivity [107].

Using QPM based on quadriwave lateral shearing interferometry (QWLSI), it was possible to follow the cytoskeleton and membrane dynamic as well as to visualize vesicles and mitochondria in living mammalian cells, directly in their culture medium with a conventional transillumination microscope equipped with a halogen lamp [108]. Actin stress fibers with a diameter up to 3.5 nm were detected with higher contrast compared to the commercial DIC [108].

**Figure 2:**
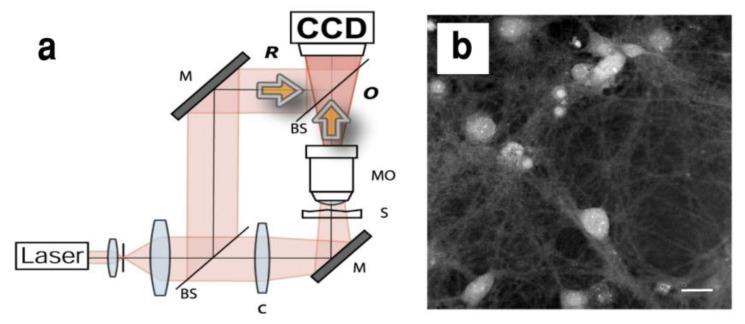
Digital holography microscopy (DHM) for exploring living neurons. (**a**) Quantitative-phase (QP)—DHM experimental setup. Beam splitter (BS), condenser (C), mirror (M), specimen (S), microscope objective (MO), object wave (O), reference beam (R) and digital CCD camera. (**b**) Representative example of quantitative-phase image of primary mouse cortical neurons, between 14 and 21 days in culture. Scale bar represents 25 μm. Reprinted with permission from Elsevier [112].

Dynamical and morphological parameters such as membrane fluctuation, intracellular protein content, cell volume, sphericity or thickness of different cell types (red blood cells, breast cancer, neutrophiles as well as neuroblastoma cells and hippocampal neurons) were recently investigated by DHM [109,110,111] with promising applications in the field of Neuroscience (Figure 2 and [112]). Transmembrane water movements during neuronal network activity, in particular during the release of neurotransmitters [93], dendritic spines dynamics (changes in size, shape and branching) [113] and neuronal cell death [89] are starting to be characterized by DHM and offer a promising tool in the diagnostics of psychiatric disorders [93]. Cytoskeleton alterations that occur in many neurodegenerative diseases [114] or cytoskeletal rearrangements required during regeneration after injury [12] could also be investigated by DHM, possibly in combination with label-based approaches to confirm the identity of the specific cytoskeletal structures involved.

One of the problems in established DHM is the diffraction limited resolution of phase measurements. A super resolution approach that determines quantitative optical properties beyond the optical diffraction limit and allows direct imaging of three-dimensional remodeling of a synaptic network with a lateral resolution of nm was proposed [115].

### 4.2. Scanning Probe Microscopy (SPM)

Scanning probe microscopy (SPM) techniques can capture nanoscale topographical dynamic changes of a cell surface under physiological conditions. Atomic force microscopy (AFM) [116] has exquisite axial resolution and can be used to obtain detailed representations of the outer membrane in three dimensions [117]. However, the forces applied by the cantilever tip deform the cell, making small, fragile structures such as microvilli or filopodia difficult to image. Additionally, the mechanosensitivity of cells, especially neurons [118], can be problematic for long-term, live cell experiments.

In contrast to AFM, scanning ion conductance microscopy (SICM) is a genuinely non-contact technique that provides high lateral and axial resolution (10 s of nanometer lateral, and less than 5 nm axial) [119,120]. Many studies have shown that due to non-invasive nature of SICM, it is an efficient method to image biological samples. Korchev [121] and Schäffer [122] have proven that SICM is particularly useful for live mammalian cell imaging. It was used for high-resolution imaging of diverse biophysical systems such as live cell dynamics [123], proteins on cell membranes [120] and suspended lipid bilayers [124]. It has been shown that SICM is particularly well-suited to study sensitive cell types such as stem cells and neurons [125]. Specifically, SICM has been used to study fixed neuronal culture [126]. However, Novak et al. used SICM to image live hippocampal neurons [125] and later Pellegrino et al. used SICM to both image and stimulate neuronal growth cones [127]. In a recent study, Takahashi et al. used SICM to visualize the dynamics of cytoskeleton changes in neurons, including dendritic spines, synapse formation and cargo transfer within dendrites and axons [126]. However, the previous time-lapse SICM imaging of neurons was below 4 h because keeping the cell viability for a long time during imaging is challenging [126].

**Figure 3:**
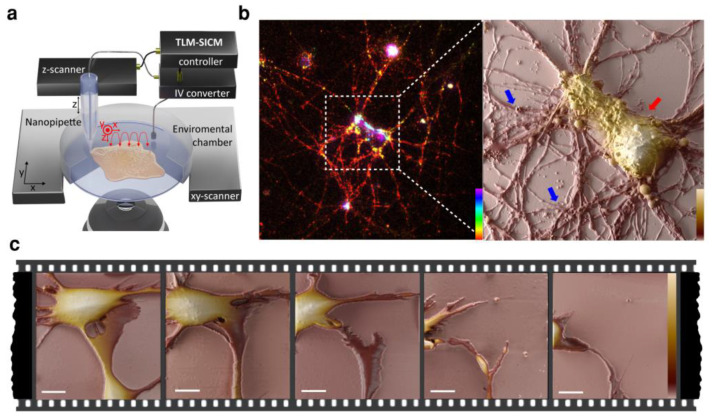
Scanning ion conductance microscopy (SICM) method for non-invasive live cell imaging at the nanoscale: (**a**) Schematic of SICM principle. Reprinted with permission under the terms of the Creative Commons CC-BY license from [128]; (**b**) Structured illumination microscopy (SIM) z-stack image of a mouse cortical neuron, fixed in 4% PFA and stained for tubulin, and SICM image on a 60 μm area. The red arrow shows the cell body and the blue arrows show the intricate network of neurites, Z scale bar: 0–8 μm. (**c**) Time-lapse live imaging of neuroblastoma cell migration using SICM, Z scale bar: 0–7.5 μm, lateral scale bar: 10 μm, temporal resolution: 20 min per image.

In a recent study, Leitao et al. developed an SICM system (Figure 3) that can perform long-term imaging of mammalian cells and follow processes up to 48 h, while keeping high-speed capabilities down to 0.5 s/frame [128]. It was also shown that SICM can be combined with fluorescence super-resolution microscopy to track the molecular activity simultaneously [128,129]. While high-speed SICM can already observe dynamic processes in neurons [126], the fragile nature of neuronal cells requires additional improvements in SICM instruments and imaging protocols. These can be tested using a more robust neuronal in vitro model such as neuroblastoma cell lines (Figure 3c). Room for improvement still exists in the achievable imaging speeds and longer time-lapse observation periods for capturing the motility of neuronal growth cones. This will require further improvements to shorten the scanning time and improve imaging conditions to maintain cell viability for a long time. The combination of SICM with live-cell-imaging-compatible super-resolution optical microscopy also holds a great deal of promise. However, as discussed earlier, the phototoxicity considerations will determine the optimal image modality. Out of the super-resolution imaging techniques available, structured illumination microscopy (SIM) could be particularly well-suited, since it has moderate light exposure while still providing a twofold resolution improvement over conventional widefield techniques [130,131]. SICM microscopes are generally built on top of inverted optical microscopes, which makes combination of SICM with fluorescent techniques relatively straightforward. Integrating SICM with the above-mentioned digital holographic microscope, on the other hand, is a more complex endeavor. If achieved, however, it would be a very potent tool for label-free long-term live cell imaging of neurons.

## 5. Conclusions

Observing living, unaltered, and unlabelled nanoscale life in action would be a uniquely powerful way to study primary cells and native tissue. Unfortunately, most existing techniques require labelling, staining, or fixation of the samples. While labelling has the advantage that specific biological structures can be separated from the complex biological background, it requires a profound intervention into the cells. To characterize the key events in many physiological processes of the nervous system, label-free and real-time live cell imaging is required. Live cell imaging is an important method to investigate the phenotype of live neuron dynamics because imaging the fixed samples does not cover the complexity of dynamic events that occur during the development and regeneration of the nervous system. Several promising new imaging approaches exist in other areas of cell biology, of which we have discussed DHM, AFM and SICM in this review (summarized in Table 1). Thus far, adoption of these advanced imaging techniques in Neuroscience is still in its infancy. However, with steadily improving instruments, model systems, and experimental procedures, the possibilities of observing neuronal development and regeneration in action is an intriguing new paradigm in Developmental Neuroscience. Embracing the capabilities made available by the development of new live cell microscopy methods will undoubtedly lead to many exciting discoveries and new lines of research.

## Figures and Tables

**Table 1:** Overview of the imaging techniques for live cell imaging of neurons. Brightfield (BF), phase contrast (PhC), differential interference contrast (DIC), Digital holography microscopy (DHM), Atomic force microscopy (AFM), Scanning ion conductance microscopy (SICM).

Imaging Technique	Resolution * (Lateral; Axial)	Acquisition Frequency * (fps)	Advantages	Limitations	References
**Label-free**					
**Transmitted light** **microscopy** **(BF, PhC, DIC)**	~200 nm; 400–700	1- over 200	Ease of use Non-invasive Long-term recordings (hours, days)	Low contrast Low resolution Phototoxicity (lower than fluorescence-base methods)	[92,93,94]
**DHM**	~260 nm; ~160–320 nm (90 nm; 150 nm with 2п-DHM)	50–160	Fast Non-invasive (low-light level of illumination intensity, ~200 μW/cm^2^) High axial sensitivity allows visualization of <10 nm structures Volumetric cell analysis	Sensitive to various sources of experimental noise	[107,108,109,112,115]
**AFM**	<10 nm	0.1–10	High resolution surface topography imaging Photobleaching- and phototoxicity-free	Resolution is dependent on the AFM tip Mechanical force induction	[116,118]
**SICM**	180 nm; <5 nm	2–4	High resolution surface topography imaging Live cell imaging in physiological conditions Photobleaching- and phototoxicity-free	Resolution is dependent on pipette Lower imaging speed compared to other methods	[118,128]
**Label-based**					
**Fluorescence** **Microscopy**	180 nm; 400 nm (widefield) ,~30 nm (superresolution imaging of live cells)	1- over 200	High signal-to-background ratio Molecular tracking In-cell imaging	Phototoxicity (several minutes at ~1 kW/cm^2^ light intensity) Photobleaching (depending on the fluorophore used) Interference of tags with cell physiology	[21,22,91,92,94]

* The data are indicative of experimental settings reported in the references and are not exhaustive, considering the rapidly evolving super-resolution methods in both fluorescence and label-free microscopy.

## Data Availability

No new data were created or analyzed in this study. Data sharing is not applicable to this article.
